# Role of the NRG1/ErbB4 and PI3K/AKT/mTOR signaling pathways in the anti-psychotic effects of aripiprazole and sertindole in ketamine-induced schizophrenia-like behaviors in rats

**DOI:** 10.1007/s10787-022-01031-w

**Published:** 2022-07-25

**Authors:** Dalia A. Nawwar, Hala F. Zaki, Rabab H. Sayed

**Affiliations:** Department of Pharmacology and Toxicology, Faculty of Pharmacy, Cairo University, Kasr El Aini St, Cairo, 11562 Egypt

**Keywords:** Aripiprazole, Ketamine, LY294002, Rats, Sertindole, Schizophrenia

## Abstract

Schizophrenia is a common mental disorder affecting patients’ thoughts, behavior, and cognition. Recently, the NRG1/ErbB4 signaling pathway emerged as a candidate therapeutic target for schizophrenia. This study investigates the effects of aripiprazole and sertindole on the NRG1/ErbB4 and PI3K/AKT/mTOR signaling pathways in ketamine-induced schizophrenia in rats. Young male Wistar rats received ketamine (30 mg/kg, intraperitoneally) for 5 consecutive days and aripiprazole (3 mg/kg, orally) or sertindole (2.5 mg/kg, orally) for 14 days. The proposed pathway was investigated by injecting LY294002 (a selective PI3K inhibitor) (25 μg/kg, intrahippocampal injection) 30 min before the drugs. Twenty-four hours after the last injection, animals were subjected to behavioral tests: the open field test, sucrose preference test, novel object recognition task, and social interaction test. Both aripiprazole and sertindole significantly ameliorated ketamine-induced schizophrenic-like behavior, as expected, because of their previously demonstrated antipsychotic activity. Besides, both drugs alleviated ketamine-induced oxidative stress and neurotransmitter level changes in the hippocampus. They also increased the gamma-aminobutyric acid and glutamate levels and glutamate decarboxylase 67 and parvalbumin mRNA expression in the hippocampus. Moreover, aripiprazole and sertindole increased the NRG1 and ErbB4 mRNA expression levels and PI3K, p-Akt, and mTOR protein expression levels. Interestingly, pre-injecting LY294002 abolished all the effects of the drugs. This study reveals that the antipsychotic effects of aripiprazole and sertindole are partly due to oxidative stress reduction as well as NRG1/ErbB4 and PI3K/AKT/mTOR signaling pathways activation. The NRG1/ErbB4 and PI3K signaling pathways may offer a new therapeutic approach for treating schizophrenia in humans.

## Introduction

Schizophrenia is a severe chronic mental disorder affecting up to 1% of the world’s population (McGrath et al. 2008). Three categories of symptoms characterize schizophrenia: positive symptoms (e.g., delusions and hallucinations), negative symptoms (e.g., social withdrawal and amotivation), and cognitive impairments (Seeman 2019). As cognitive impairment is a core feature of schizophrenia, there is an urgent need to develop new therapeutic strategies that focus on improving these symptoms (Hou et al. 2020).

Although the pathophysiology of schizophrenia is unclear, several assumptions and hypotheses have arisen. Accumulating evidence suggests that *N*-methyl-d-aspartate (NMDA) receptor hypofunction plays a role in the pathogenesis of schizophrenia (Weickert et al. 2013; Nakazawa et al. 2017). Indeed, NMDA receptor antagonists, such as phencyclidine and ketamine, show behavioral symptoms in rodent models and humans that resemble both positive and negative schizophrenia symptoms (Krystal 1994; Neill et al. 2010) and also induce oxidative damage to brain lipids, proteins, and DNA which in turn impair cell function and viability (Zhang et al. 2006; de Oliveira et al. 2009)..

Neuregulin1 (NRG1), a growth factor that binds and activates ErbBs (transmembrane tyrosine kinase receptors), plays a critical role in neurotransmission and synaptic plasticity in the cortex and hippocampus (Mei and Nave 2014). ErbB4 is the only receptor with a high affinity for NRG1 (Buonanno 2010). NRG1/ErbB4 signaling is strongly linked to working memory dysfunction, and several recent studies have investigated its relevance as a candidate therapeutic pathway for schizophrenia (Yamazaki and Sumikawa 2017; Chung et al. 2018; Hasan et al. 2020). The loss of ErbB4 signaling decreases the excitatory synaptic inputs to parvalbumin (PV) interneurons, reducing their activity and subsequently lowering activity-dependent PV expression and impairing working memory (Ting et al. 2011; delPino et al. 2013; Chung et al. 2018). ErbB4 activation by NRG1 causes the phosphorylation of phosphatidylinositol-3-kinase (PI3K) and its activation (Mei and Xiong 2008). PI3K phosphorylates and activates AKT, which activates the mammalian target of rapamycin (mTOR), among other downstream effects (Polak and Hall 2009).

The role of NRG1/ErbB4 in schizophrenia is controversial. Experimental studies showed that both downregulating (Rimer et al. 2005; O’Tuathaigh et al. 2007; Bian et al. 2009; Van Den Buuse et al. 2009) and upregulating (Deakin et al. 2009; Kato et al. 2010) NRG1–ErbB4 signaling-induced schizophrenia-like abnormal behavior. However, postmortem studies revealed an increased NRG1/ErbB4 expression or increased ErbB4 receptor phosphorylation in psychotic patients (Hashimoto et al. 2004; Hahn et al. 2006; Law et al. 2006; Chong et al. 2008). Recently, study by Yang et al. (2022) showed that antipsychotic drugs increase NRGβ1 serum levels in schizophrenia patients with suggestions for improving the treatment of psychotic symptoms.

Atypical antipsychotics have a wider range of benefits for improving cognitive deficits than typical antipsychotics (Meltzer and McGurk 1999; Harvey and Keefe 2001; Keefe et al. 2004; Hou et al. 2020). Aripiprazole and sertindole are atypical antipsychotics that function as antagonists of both the serotonin and postsynaptic dopamine (DA) D2 receptors (Gupta and Masand 2004; Kasper et al. 2010). Aripiprazole and sertindole alleviate both positive and negative symptoms of schizophrenia and partially improve cognitive dysfunction without producing extrapyramidal side effects (Gupta and Masand 2004; Perquin and Steinert 2004; Hereta et al. 2020). Although these drugs improve cognitive function in patients with schizophrenia, their mechanism of action remains unclear. Thus, this study aimed to investigate the effects of aripiprazole and sertindole on the NRG1/ERbB4 and PI3K/AKT/mTOR signaling pathways in ketamine-induced schizophrenia in rats.

## Materials and methods

### Animals

Young male Wistar rats weighing 100–120 g were obtained from the animal facility of the Faculty of Pharmacy, Cairo University (Cairo, Egypt). Animals were housed them under standard conditions: 60% ± 10% humidity, room temperature (25℃ ± 2℃), and a 12/12-h light/dark cycle. Animals had ad libitum access to food and water. The Ethics Committee for Animal Experimentation of the Faculty of Pharmacy, Cairo University, approved the study protocol (Permit Number: PT 2110). We followed the Guide for Care and Use of Laboratory Animals published by the US National Institutes of Health (Publication No. 85-23, revised 2011) for all the experiments. All efforts were made to minimize the number of rats used and their suffering.

### Drugs and chemicals

Aripiprazole, sertindole, and ketamine were purchased from Chemipharm Pharmaceutical Industries (Sixth October City, Egypt), H. Lundbeck A/S (Copenhagen, Denmark), and Trokiaa Pharmaceuticals Ltd. (Ahmedabad, India), respectively. Aripiprazole and sertindole were dissolved in a minimum amount of 0.1-M hydrochloric acid and diluted with saline, and then administered (1 ml/200 g of body weight) orally (p.o.). Ketamine was diluted in normal saline and administered (0.1 ml/200 g of body weight) intraperitoneally (i.p.). PI3K inhibitor, LY294002 hydrochloride, was purchased from Sigma-Aldrich Chemical Co. (St. Louis, MO, USA) and dissolved it in 1% DMSO. All other chemicals were of the highest commercially available purity grade.

### Experimental design

**Fig. 1:**
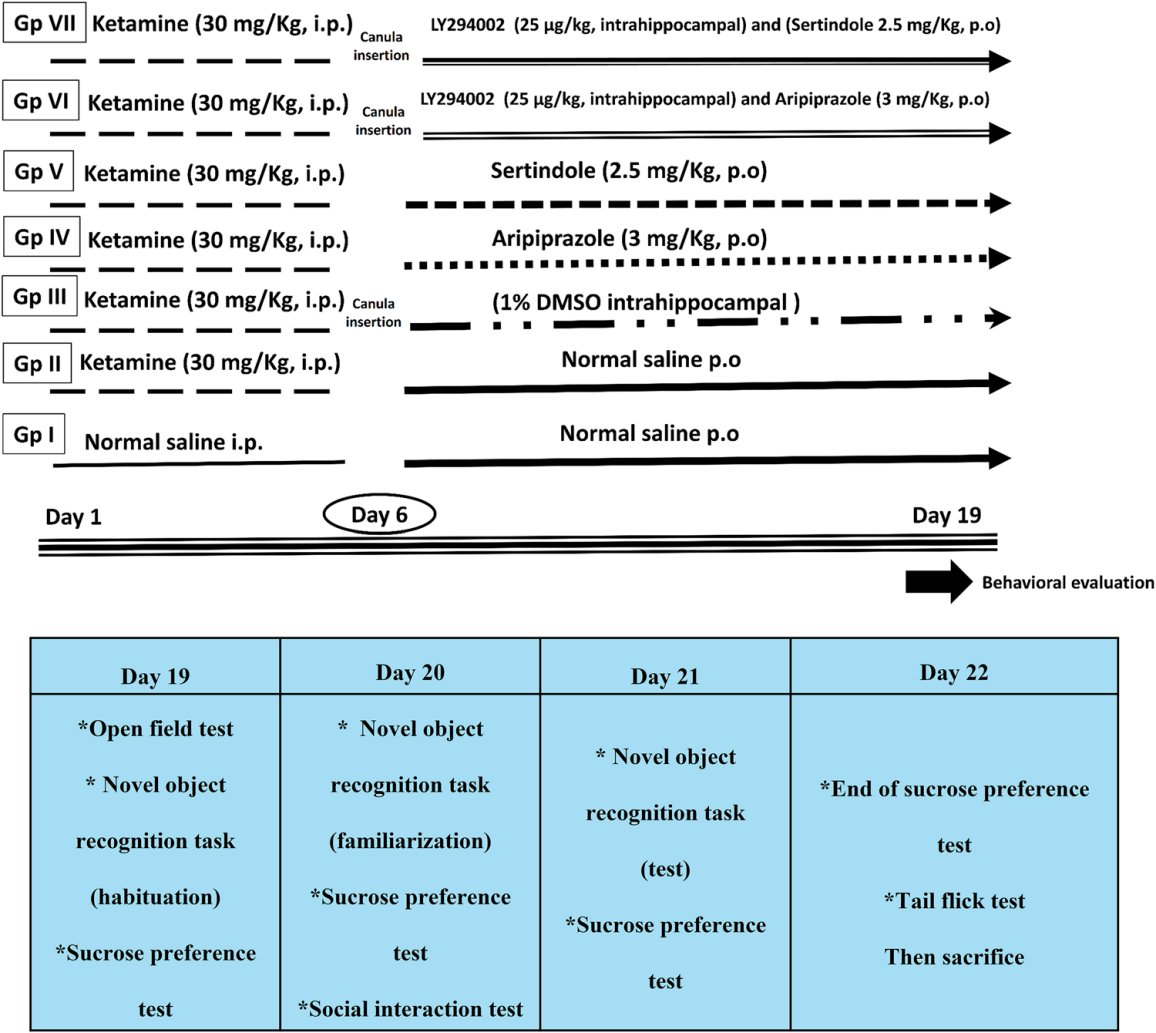
Experimental design

A technical assistant who was not involved in the analysis divided 140 rats randomly into seven groups (*n* = 20), as depicted in Fig. 1. Group I (Control): rats received normal saline i.p. for 5 consecutive days and then p.o. for 14 days and served as the baseline control group. Group II (Ket): rats received ketamine (30 mg/kg, i.p.) (Lisek et al. 2016) for 5 consecutive days and then normal saline p.o. for 14 days. Group III (Ket + DMSO): rats received ketamine (30 mg/kg, i.p.) for 5 consecutive days and then a daily intrahippocampal 1% DMSO injection for 14 days. Group IV (Ket + Arp): rats received ketamine for 5 days and then aripiprazole (3 mg/kg, p.o.) (Carli et al. 2011) for 14 days. Group V (Ket + Ser): rats received ketamine for 5 days and then sertindole (2.5 mg/kg, p.o.) (Idris et al. 2010) for 14 days. Group VI (Ket + LY + Arp): rats received ketamine and then LY294002 (25 μg/kg, intrahippocampal) (Hongyan et al. 2017) and aripiprazole for 14 days. Group VII (Ket + LY + Ser): rats received ketamine and then LY294002 and sertindole for 14 days. A unilateral intrahippocampal injection of LY294002 was done in a volume of 2 µl 30 min before the anti-psychotic.

After the last injection, we subjected the animals to behavioral tests: the open field test, sucrose preference test, novel object recognition task, and social interaction test. We also performed the tail flick test to ensure that any differences observed during the behavioral tests were not due to changes in nociception (i.e., analgesia). We conducted the behavioral tests between days 19 and 22 of the experiment and arranged them in sequence from the least to the most stressful tests.

After the behavioral tests, we divided the rats in each group into four sets and killed them by cervical dislocation under light anesthesia. We then rapidly dissected the brains and washed them with ice-cold saline. In the first set (*n* = 2), we fixed the brains with 10% (v/v) formalin for 24 h to perform histopathological staining with hematoxylin and eosin (H&E). In the other sets, we promptly dissected the hippocampi from the injected site and stored them at −80℃. We used the hippocampi of the second set (*n* = 6) to estimate malondialdehyde (MDA) and glutathione (GSH) levels. We used the hippocampi of the third set (*n* = 6) to quantify the DA, glutamate, and gamma-aminobutyric acid (GABA) levels using enzyme-linked immunosorbent assays (ELISA), and phospho-PI3K, phospho-AKT, and mTOR protein expression by western blot analysis. Finally, we used the hippocampi of the fourth set (*n* = 6) to quantify NRG1, ERbB4, glutamate decarboxylase 1 (GAD67), and PV mRNA expression using real-time quantitative polymerase chain reaction (PCR).

### Unilateral guide cannulae implantation

We anesthetized the rats with thiopental (50 mg/kg, i.p.) (EPICO, Cairo, Egypt) and placed them in a stereotaxic frame (David Kopf Instruments, CA, USA). Next, we exposed the bregma and drilled a small hole for unilateral right intrahippocampal implantation of a 23-gauge, stainless steel guide cannula using the following stereotaxic coordinates: −4.3 mm posterior to bregma, ±2.4 mm lateral to the midsagittal suture, and −2.6 mm ventral from the brain surface (Vallée et al. 1997; Paxinos and Watson 2004). We then fixed the guide cannulae using dental cement (Durelon; Thompson Dental Supply, Raleigh, NC, USA). After the surgical procedure, we housed the rats individually and administered them 30,000 U of penicillin G (Durapen; GC Hanford, New York, NY, USA) intramuscularly in aqueous suspension, followed by LY294002 or 1% DMSO using a 30-gauge injector. We left the needle tip in place for 2 min to avoid drug leakage along the needle track.

### Behavioral assays

In order to mitigate selection bias, rats were randomly selected by other researcher for the following behavioral tests.

#### Open field test

We used an 80 × 80 × 40 cm square wooden box with red painted walls and white lines dividing the floor into a 4 × 4 grid of 16 equal squares. We cleaned and dried the floor of the apparatus after each measurement. The test took place under a dim white light in a quiet room. We placed each rat in the center of the box and allowed it to explore the apparatus for 5 min. Then, we video-recorded locomotor activity for 5 min. We evaluated ambulation (number of squares crossed), rearing (frequency of standing on the hind legs), and latency time (time spent immobile; Walsh and Cummins 1976).

#### Sucrose preference test

We placed the rats in individual cages with free access to food for 3 days. Each rat had access to two identical bottles placed randomly in the cage (we switched the position of the bottles every day), one containing 250 ml of tap water and the other 250 ml of 1% w/v sucrose in tap water. We measured the remaining volume in each bottle at the end of each day. We calculated sucrose preference as the ratio of sucrose intake to total fluid intake and expressed the values as percentages. The loss preference for sweetened water over regular water indicates anhedonic behavior, an important negative symptom (Tandon et al. 2009).

#### Novel object recognition task

We performed the test as described previously (Ennaceur and Delacour 1988; Boultadakis and Pitsikas 2010). We used a dark open wooden box (80 cm in length, 50 cm in width, and 60 cm in height). The light intensity was distributed equally in the box. It contained distinguishable objects made of wood, in two different shapes (cubes and pyramids) and colors, each 7 cm high. The rats could not move the objects. The animals had 3 min to discover the empty arena and then 3 min to inspect two identical objects. On the next day, we substituted one of the identical objects with a different object. Next, we placed the rats individually in the center of the arena and left them for 3 min with the two different objects located in opposite corners: the familiar (F) and the novel object (N). We recorded the time that the rats spent inspecting each object. After each measurement, we cleaned the arena and objects carefully with 70% ethanol to avoid bias due to the odor left by the previous rat. We calculated the discrimination index (DI) to assess the preference for N or F as follows: DI = N − F/N + F. Moreover, we calculated the preference index (PI) as the percentage of time that the rats spent discovering the N object, relative to the total time spent by the rat discovering both objects, as follows: PI = N/(N + F] × 100 (%) (Cavoy and Delacour 1993).

#### Social interaction test

Compromised social interaction is a characteristic behavior in schizophrenia animal models (Mohn et al. 1999). We performed the test using an open field test apparatus. We housed the animals individually 3 h before the test. For the task, we placed two animals of the same group into a cage for 7 min. We evaluated the social behavior of each pair of animals by measuring the latency to the first interaction, the number of social contacts, and total time of contacts (Niesink and Van Ree 1989; Schneider and Przewłocki 2005).

#### Tail flick test

In a circular glass bowl, we heated 1800 ml of water to 55℃ and kept the bowl on the heating plate to maintain the water temperature during the experiment. Next, we placed 2–3 cm of the tip of the rat’s tail in the water and recorded the time until the rat flicked its tail out of the water (Bolton et al. 2012).

### Biochemical parameters

#### Quantification of oxidative stress biomarkers

We estimated hippocampal lipid peroxidation by measuring the MDA level. We quantified MDA by measuring the thiobarbituric acid-reactive substances (Ohkawa et al. 1979) using a colorimetric assay kit (Biodiagnostic, Dokki, Giza, Egypt). We expressed the results in nmol/mg protein. We quantified the hippocampal reduced GSH content spectrophotometrically using Ellman’s reagent (Beutler et al. 1963). We expressed the results in mmol/mg protein.

#### Enzyme-linked immunosorbent assay (ELISA)

We estimated the hippocampal DA and GABA levels using rat ELISA kits purchased from MyBiosource Inc. (San Diego, CA, USA). Similarly, we quantified hippocampal glutamate levels using rat ELISA kits (Cell Biolabs Inc., San Diego, CA, USA). We followed the manufacturer’s instructions for all the procedures. We expressed the results in ng/mg protein for DA and glutamate and in pg/mg protein for GABA. We measured the protein content following the method described by Lowry et al. (1951).

#### Real-time quantitative polymerase chain reaction

We extracted total RNA from hippocampal tissues using the SV Total RNA Isolation System (Promega, Madison, WI, USA) and confirmed the purity of the obtained RNA spectrophotometrically by measuring the optical density at 260/280 nm. According to the manufacturer’s instructions, we reverse-transcribed equal amounts of extracted RNA into cDNA using a reverse transcription kit (Fermentas, Massachusetts, USA). To assess NRG1, ERbB4, GAD67, and PV gene expression, we performed quantitative RT-PCR using an applied biosystem with software version 3.1 (StepOne™, USA). Briefly, we added 5 μl of cDNA to 12.5 μl of SYBR Green mixture, 5.5 μl of RNase-free water, and 1 μl of each primer (total volume: 25 µl). Table 1 shows the sequences of the primers. The PCR amplification consisted of 40 cycles of denaturation for 15 s at 95℃, annealing for 60 s at 60℃, and extension for 60 s at 72℃. After the quantitative RT-PCR run, we obtained the relative expression of the target gene using the 2^−ΔΔCT^ formula (Pfaffl 2001). We used β-actin to normalize the mRNA levels.

**Table 1:** Primer sequences used for RT-PCR

Gene	Forward primer (5′ → 3′)	Reverse primer (5′ → 3′)
NRG1	TGATCGTTGCCAAAACTACG	ACCAACAGGGCGATACAGAT
ERbB4	TGCCATAAGTCTTGCACTGG	CGTAGGGTCCATAGCACCTG
GAD67	CAAGTTCTGGCTGATGTGGA	GCCACCCTGTGTAGCTTTTC
PV	AAGAGTGCGGATGATGTGAAG	AGCCATCAGCGTCTTTGTTT
β-actin	CGTTGACATCCGTAAAGACCTC	TAGGAGCCAGGGCAGTAATCT

#### Western blot analysis

After extracting the total protein from the hippocampus tissue, we loaded equal amounts of proteins onto a sodium dodecyl sulfate–polyacrylamide gel electrophoresis lane to separate them by molecular weight. Next, we transferred the proteins to a polyvinylidene difluoride membrane using a Trans-Blot® Turbo™ Transfer System (Bio-Rad Laboratories GmbH, München, Germany). We then soaked the membranes in Tris-buffered saline with Tween 20 buffer and 3% bovine serum albumin at room temperature for 1 h to block nonspecific binding sites. Next, we developed the blots using antibodies against phospho-PI3K (Tyr607) (MBS8511424), phospho-AKT (Thr308) (MBS855182), mTOR, and β-actin obtained from (Thermo Fisher Scientific Inc., Rockford, IL, USA). After washing, we added horseradish peroxidase–conjugated antibodies. Finally, we developed the blots using enhanced chemiluminescence substrate (Bio-Rad Laboratories Inc., Hercules, CA, USA). We assessed the band intensity of the target proteins against the control sample after normalization relative to β-actin using image analysis software and expressed the results as arbitrary units.

### Histopathological examination

We flushed the brain samples and fixed them in 10% neutral buffered formalin for 72 h. We trimmed and processed the samples in serial grades of alcohol and then cleared them in xylene. We filtered the samples and embedded them in paraplast tissue-embedding media. We cut 4-μm-thick coronal brain sections using a rotatory microtome. We stained the sections with H&E and then examined them through a Full HD microscopic camera operated by Leica application software (Leica Microsystems GmbH, Wetzlar, Germany).

### Statistical analyses

We checked normality using the Kolmogorov–Smirnov test. We expressed data as the means ± standard deviations. We analyzed the datasets using one-way analysis of variance, followed by the Tukey–Kramer multiple comparison test. We used GraphPad Prism software (version 6; GraphPad Software, Inc., San Diego, CA, USA) to carry out the statistical analyses and make the graphs. We set the significance level to *p* < 0.05 for all statistical tests.

## Results

As the two control groups (Ket and Ket + DMSO) were statistically identical, we used the Ket group for statistical comparisons.

### Effects of aripiprazole and sertindole on ketamine-induced behavioral changes

**Fig. 2:**
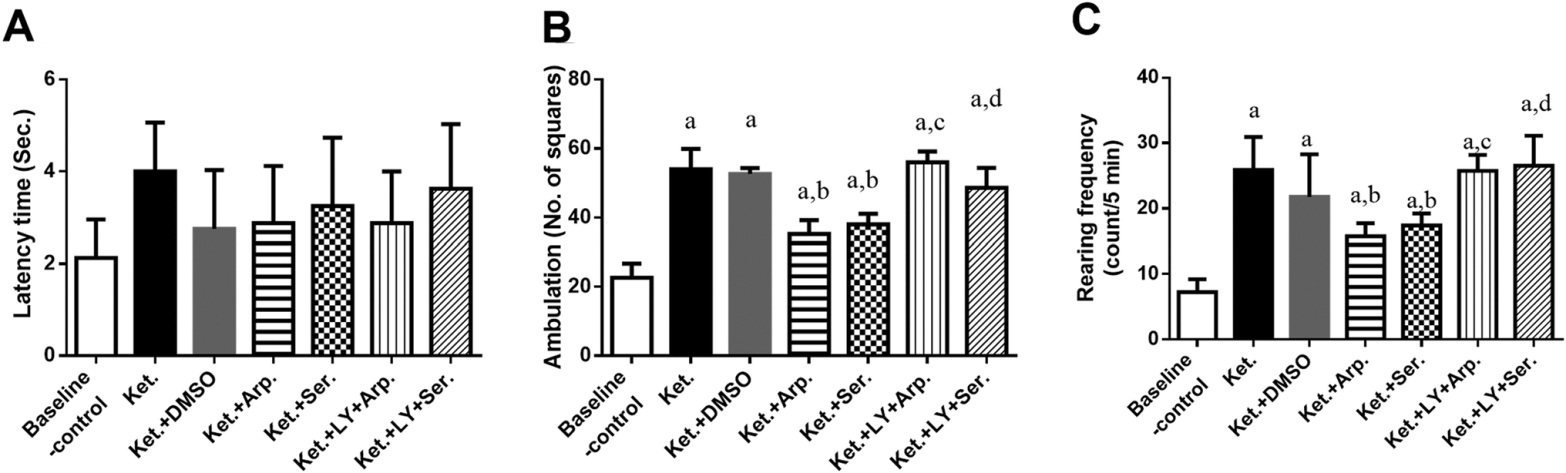
Effects of aripiprazole or sertindole on open field latency (**A**) (F (6, 49) = 2.024, *P = *0.0802), ambulation frequency (**B**) (F (6, 49) = 68.50, *P* < 0.0001), and rearing frequency (**C**) (F (6, 49) = 26.21, *P* < 0.0001) in ketamine-induced schizophrenia in rats either alone or with the PI3K inhibitor LY294002. Each bar with a vertical line represents the mean of experiments ± S.D. (*n* = 8). a vs. the baseline control group, b vs. ketamine-treated rats, c vs. the aripiprazole-treated group, and d vs. the sertindole-treated group. Statistical analyses were performed using ANOVA followed by Tukey’s post hoc test, and the criterion for statistical significance was set to *p* < 0.05

Figure 2 shows the open field test results. The Ket group had significantly higher ambulation and rearing frequencies than the baseline control group (by 145 and 255%, respectively). However, aripiprazole and sertindole significantly ameliorated ketamine-induced locomotor activity impairments, as the ambulation frequencies of the Ket + Arp and Ket + Ser groups were, respectively, 35 and 30% lower than that of the Ket group, and their rearing frequencies were, respectively, 40 and 32% lower than that of the Ket group. Pretreatment with the PI3K inhibitor LY294002 completely blocked the effects of aripiprazole and sertindole on the open field test parameters.

**Fig. 3:**
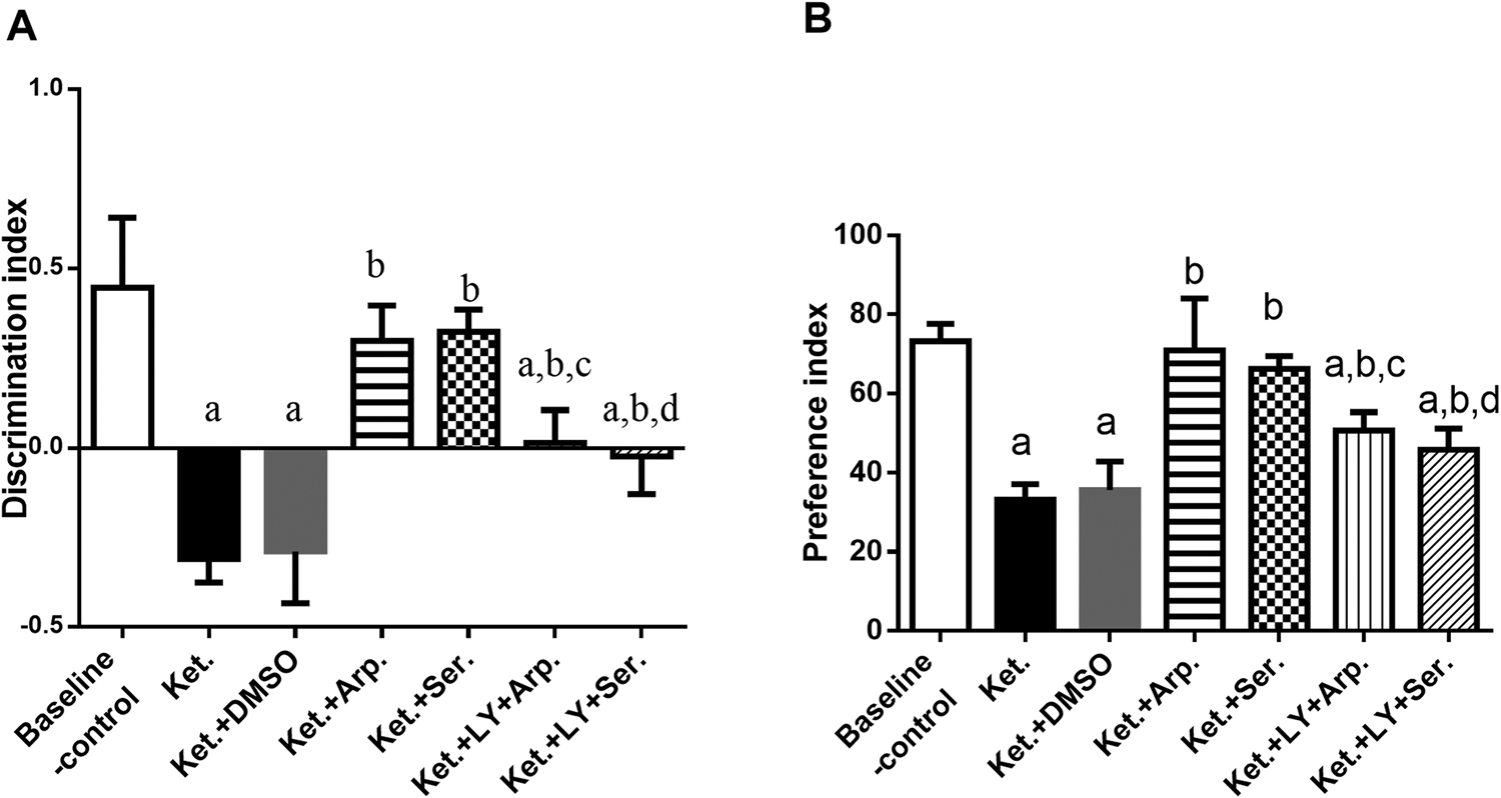
Effects of aripiprazole or sertindole on novel object recognition test, discrimination index (DI) (**A**) (F (6, 49) = 52.56, P < 0.0001), and preference index (PI) (**B**) (F (6, 49) = 47.95, P < 0.0001), in ketamine-induced schizophrenia in rats either alone or with the PI3K inhibitor LY294002. Each bar with a vertical line represents the mean of experiments ± S.D. (*n* = 8). a vs. the baseline control group, b vs. ketamine-treated rats, c vs. the aripiprazole-treated group, and d vs. the sertindole-treated group. Statistical analyses were performed using ANOVA followed by Tukey’s post hoc test, and the criterion for statistical significance was set to *p* < 0.05

Figure 3 demonstrates the ketamine-induced cognitive deficits in the novel object recognition task. Ketamine reduced the DI and decreased the PI by more than half, indicating that the rats could not discriminate between the familiar and novel objects as compared to the baseline control group. However, aripiprazole and sertindole restored this ability. Besides, groups that received LY294002 along with the antipsychotics had only moderately better DI and PI than the Ket group.

**Fig. 4:**
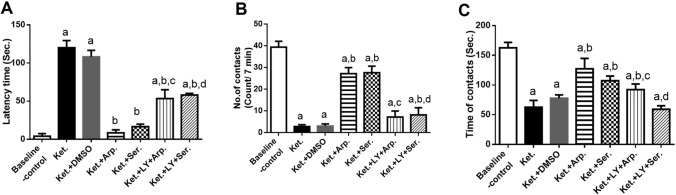
Effects of aripiprazole or sertindole on social interaction test, latency time (**A**) (F (6, 28) = 224.8, *p* < 0.0001), No. of contacts (**B**) (F (6, 28) = 169.1, *p* < 0.0001), and time of contacts (**C**) (F (6, 28) = 65.15, *p* < 0.0001) in ketamine-induced schizophrenia in rats either alone or with the PI3K inhibitor LY294002. Each bar with a vertical line represents the mean of experiments ± S.D. (*n* = 5). a vs. the baseline control group, b vs. ketamine-treated rats, c vs. the aripiprazole-treated group, and d vs. the sertindole-treated group. Statistical analyses were performed using ANOVA followed by Tukey’s post hoc test, and the criterion for statistical significance was set to *p* < 0.05

Similarly, the social interaction test showed that the Ket group had significantly higher onset latency, fewer contacts with each other, and shorter total interaction duration than the baseline control group. Besides, aripiprazole and sertindole normalized the onset latency and significantly increased the number of contacts and total interaction duration, and LY294002 reduced these improvements (Fig. 4).

**Fig. 5:**
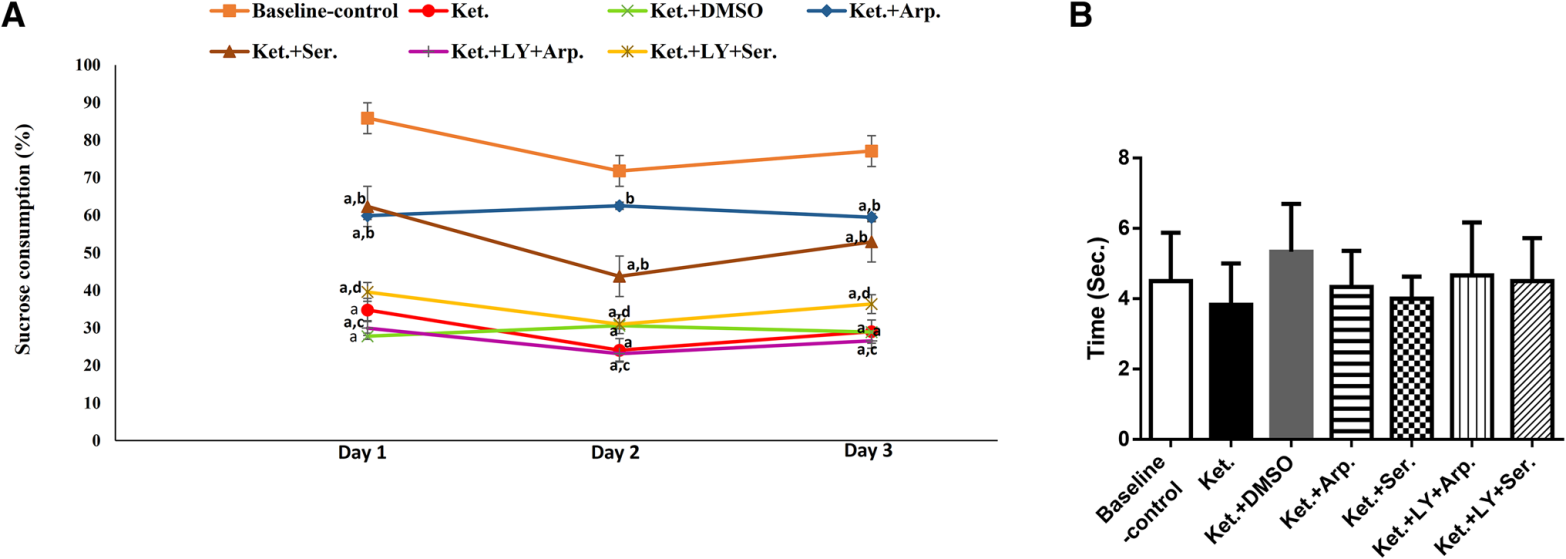
Effects of the aripiprazole or sertindole treatments on sucrose preference test (**A**) (F (6, 28) = 224.8, *p* < 0.0001) and tail flick test (**B**) (F (6, 35) = 0.9646, *p* = 0.4632) in ketamine-induced schizophrenia in rats either alone or with the PI3K inhibitor LY294002. Each bar with a vertical line represents the mean of experiments ± S.D. (*n* = 6). a vs. the baseline control group, b vs. ketamine-treated rats, c vs. the aripiprazole-treated group, and d vs. the sertindole-treated group. Statistical analyses were performed using ANOVA followed by Tukey’s post hoc test, and the criterion for statistical significance was set to *p* < 0.05

The sucrose preference of the Ket group was lower than that of the baseline control group by 40%, and aripiprazole and sertindole increased it again by 70 and 66% compared to that of the baseline control group, respectively. Again, LY294002 blocked the beneficial effects of the antipsychotics. Finally, ketamine did not significantly affect tail flick latency (Fig. 5).

### Effects of aripiprazole and sertindole on ketamine-induced histopathological alterations

**Fig. 6:**
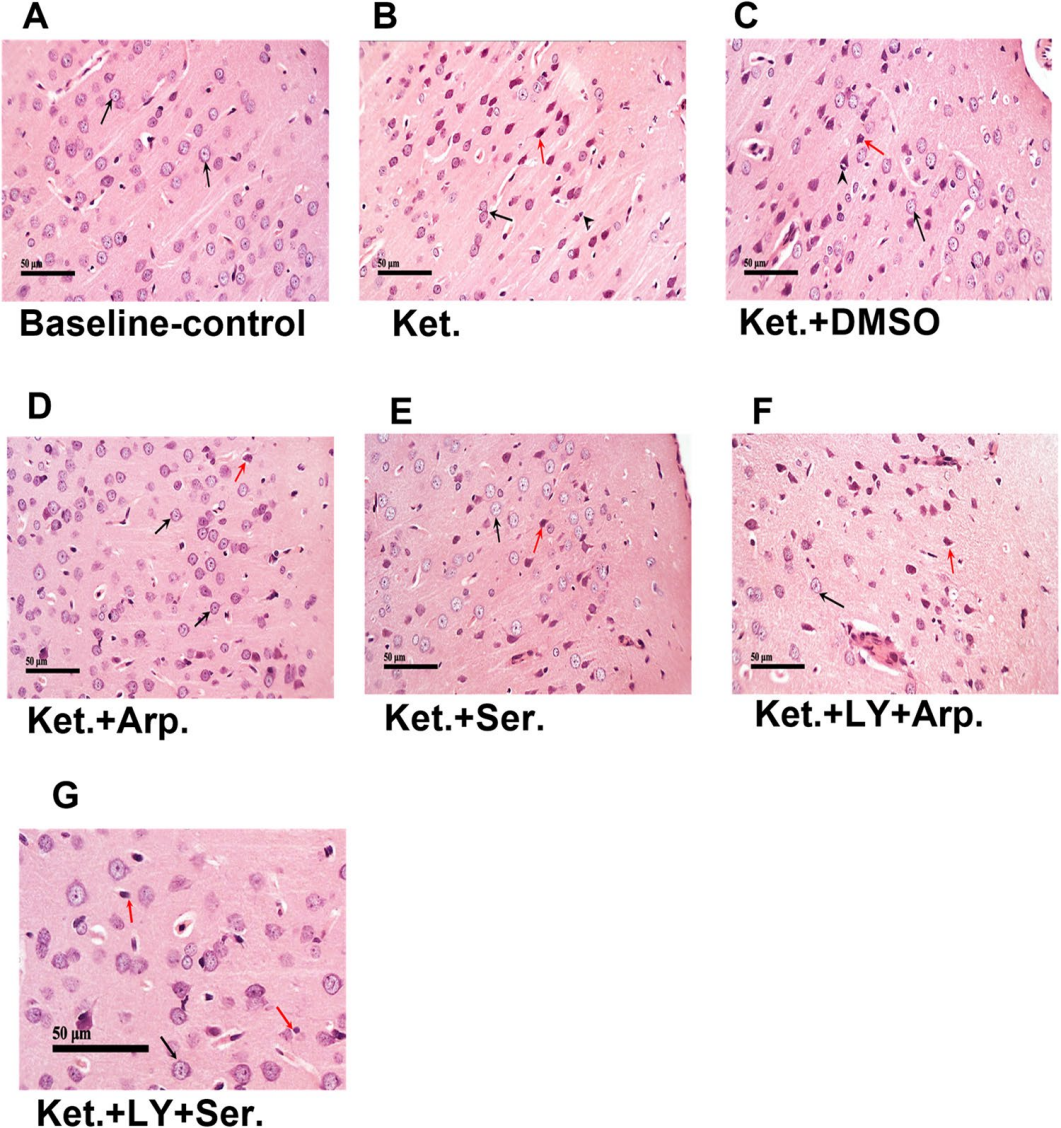
Representative photomicrographs showing the effect of the aripiprazole or sertindole on cortex in ketamine-induced schizophrenia in rats either alone or with the PI3K inhibitor LY294002. **A** (baseline control) demonstrated normal morphological features of cerebral cortex layers with apparent intact neurons with intact cellular details (Arrow), intact intercellular tissue, and minimal glial cells infiltrations. **B**, **C** (Ket and Ket + DMSO) showed focal areas of moderate neuronal damage with dark shrunken cell bodies and pyknotic nuclei (red arrow) in outer cortical layers alternated with apparent intact neurons (black arrow) and mild glial cells infiltrates (arrow head). **D** (Ket + Arp) showed almost intact cerebral cortex with many intact neurons (black arrow) and minimal records of degenerated neurons (red arrow). **E** (Ket + Ser) demonstrated more apparent intact neurons (black arrow) all over outer cortical layers alternated with fewer dispersed degenerated neurons in-between (red arrow) and mild glial cells infiltrates. **F** (Ket + LY + Arp) showed many focal areas of neuronal loss with few intact neurons (black arrow) alternated with many degenerated shrunken neurons (red arrow) and mild glial cells infiltrates. **G** (Ket + LY + Ser) showed some intact neurons (black arrow) and various degenerated neurons (red arrow)

Sections from baseline control rats revealed normal histological structures of neurons in the cerebral cortex (Fig. 6A). By contrast, ketamine-treated rats had focal areas of moderate neuronal damage, with dark, shrunken cells and mild glial cell infiltrates in the outer cortical layer (Fig. 6B, C). Both aripiprazole and sertindole ameliorated these histopathological changes: only a few neurons showed degeneration (Fig. 6D, E), whereas the coadministration of LY294002 antagonized their beneficial effects (Fig. 6F, G).

**Fig. 7:**
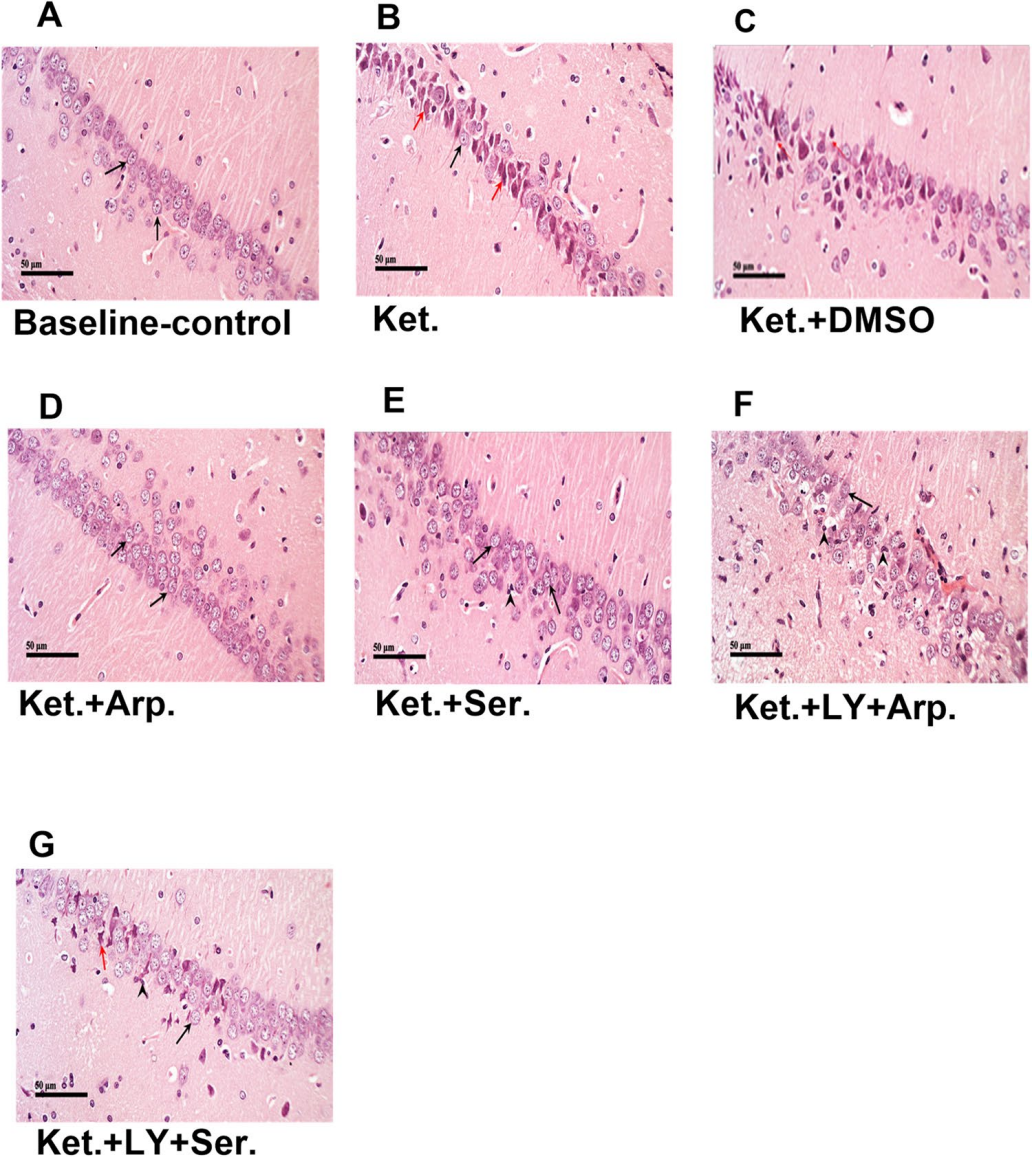
Representative photomicrographs showing the effect of the aripiprazole or sertindole on hippocampal CA1 in ketamine-induced schizophrenia in rats either alone or with the PI3K inhibitor LY294002. **A** (baseline control) demonstrated normal morphological features of hippocampal layers including intact pyramidal neurons (black arrow) with intact cellular details. **B** (Ket) showed higher numbers of degenerated shrunken pyramidal neurons (red arrow) and fewer apparent intact one (black arrow) with mild glial cells infiltrates. **C** (Ket + DMSO) showed moderate neuronal damage with alternated areas of intact (black arrow) or degenerated neurons (red arrow). **D** (Ket + Arp) showed intact hippocampal CA1 layers with apparent intact pyramidal neurons (black arrow) without abnormal morphological alterations. **E** (Ket + Ser) showed almost intact neurons without abnormal cellular alterations in CA1 layer (black arrow) and mild higher glial cells infiltrates. **F** (Ket + LY + Arp) showed many intact neuronal cell bodies (black arrow) with moderate perineuronal edema and obvious higher glial cells infiltrates (arrow head). **G** (Ket + LY + Ser) showed mild neuronal damage with few dark, shrunken, and pyknotic pyramidal cells (red arrow) interspersed between more apparent intact neurons (black arrow).

**Fig. 8:**
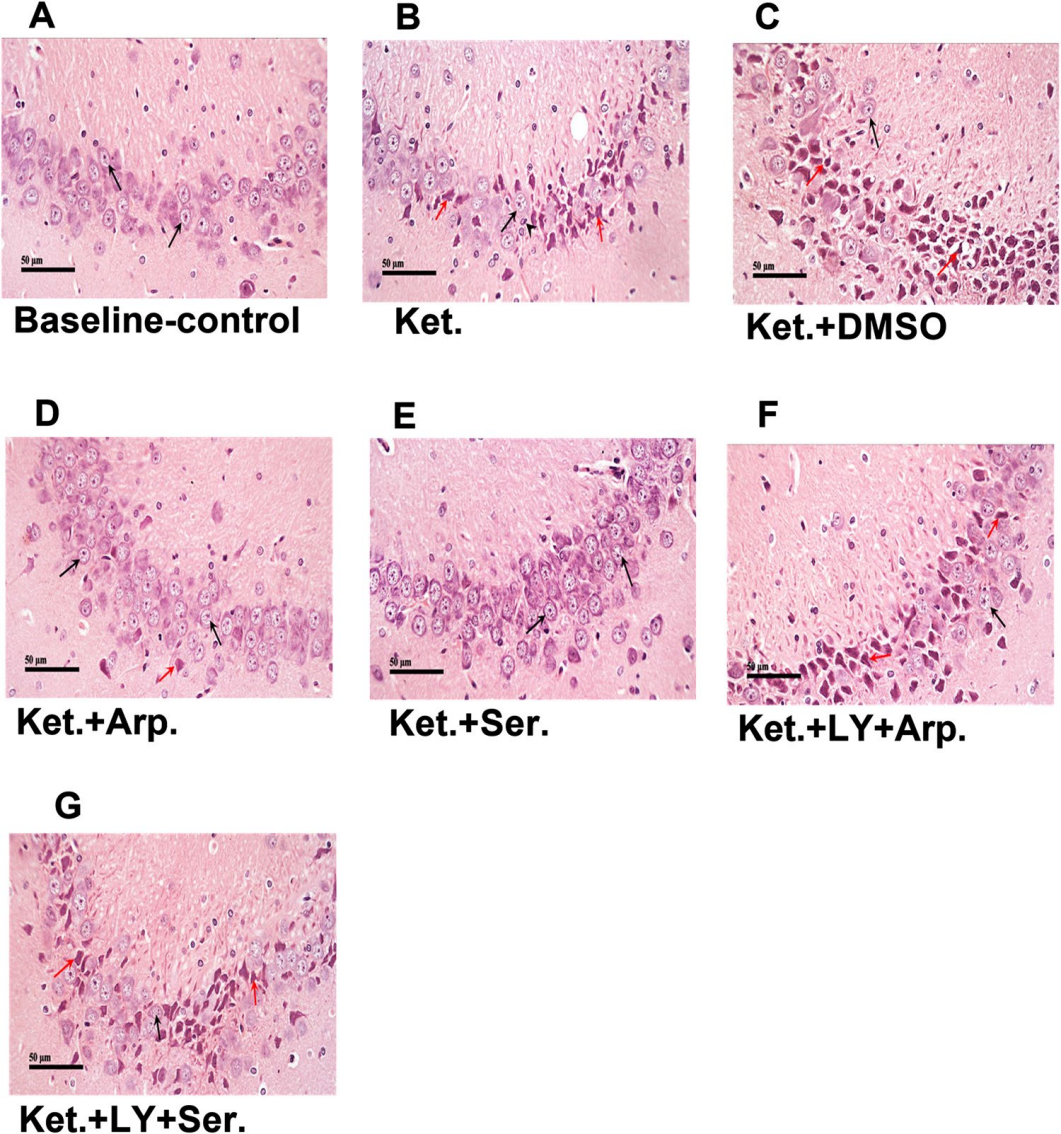
Representative photomicrographs showing the effect of the aripiprazole or sertindole on hippocampal CA3 in ketamine-induced schizophrenia in rats either alone or with the PI3K inhibitor LY294002. **A** (baseline control) demonstrated normal morphological features of hippocampal layers including intact pyramidal neurons (black arrow) with intact cellular details. **B** (Ket) showed moderate to severe neuronal damage and loss with many dark, shrunken and pykotic pyramidal cells (red arrow) alternated with fewer apparent intact neurons (black arrow). Higher glial cells infiltrate (arrow head). **C** (Ket + DMSO) showed sever neuronal damage and loss with many degenerated or necrotic neurons (red arrow) alternated with few intact cells (black head), moderate perineuronal and intercellular edema. **D** (Ket + Arp.) showed apparent intact hippocampal CA3 layers with many apparent intact pyramidal neurons (black arrow) and few scattered records of degenerated neurons (red arrow). **E** (Ket + Ser) showed almost intact neurons without abnormal cellular alterations in CA3 layer (black arrow). **F**, **G** (Ket + LY + Arp) (Ket + LY + Ser) showed severe neuronal damage with alternated apparently intact (black arrow) or degenerated, shrunken, and pyknotic neurons (red arrow).

Similarly, sections from the baseline control group revealed a normal histological appearance and distribution of the neuronal cells in the cornu ammonis 1 (CA1) (Fig. 7A) and cornu ammonis 3 (CA3) (Fig. 8A). By contrast, ketamine-treated rats had extensive neuronal degeneration, nuclear pyknosis, and gliosis in the CA1 (Fig. 7B) and CA3 (Fig. 8B). Both aripiprazole and sertindole reduced the ketamine-induced neurological damage in CA1 and CA3, as evidenced by the apparently intact neurons in the Ket + Arp group and the mild neuronal degenerative changes observed in the Ket + Ser group. The coadministration of LY294002 reversed the protective effects of the antipsychotics on ketamine-induced hippocampal neuronal injury (Figs. 7 and 8).

### Effects of aripiprazole and sertindole on ketamine-induced oxidative stress

**Fig. 9:**
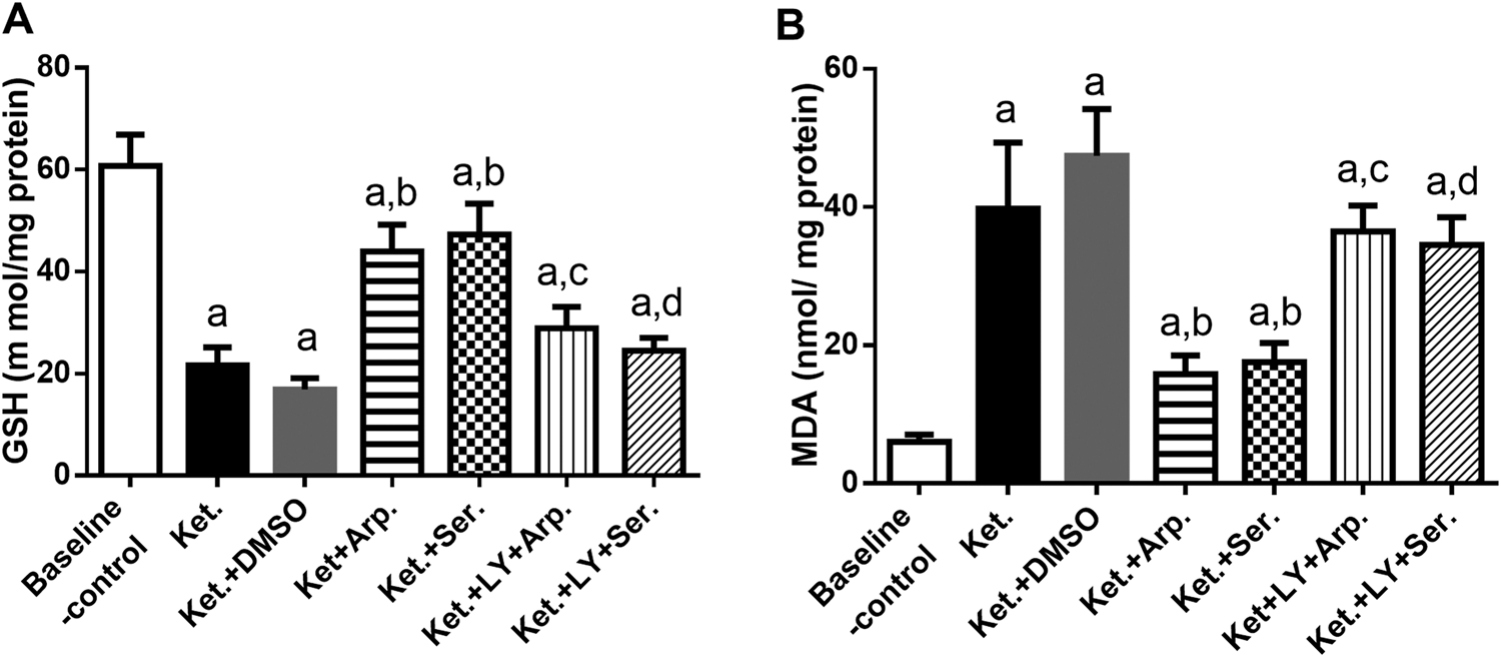
Effects of aripiprazole on sertindole treatments on GSH (**A**) (F (6, 35) = 75.39, *p* < 0.0001) and MDA (**B**) (F (6, 35) = 53, *p* < 0.0001), in ketamine-induced schizophrenia in rats either alone or with the PI3K inhibitor LY294002. Each bar with a vertical line represents the mean of experiments ± S.D. (*n* = 6). a vs. the baseline control group, b vs. ketamine-treated rats, c vs. the aripiprazole-treated group, and d vs. the sertindole-treated group. Statistical analyses were performed using ANOVA followed by Tukey’s post hoc test, and the criterion for statistical significance was set to *p* < 0.05

In this study, ketamine administration induced oxidative stress in the hippocampus of rats, as indicated by the marked increase in MDA level and the noticeable reduction in GSH level as compared with the baseline control group. Conversely, both aripiprazole and sertindole increased the GSH level by almost twofold and decreased MDA by approximately 60 and 55%, respectively. LY 294002 reversed the effects of the antipsychotics on MDA and GSH levels (Fig. 9).

### Effects of aripiprazole and sertindole on ketamine-induced changes in hippocampal neurotransmitters

**Fig. 10:**
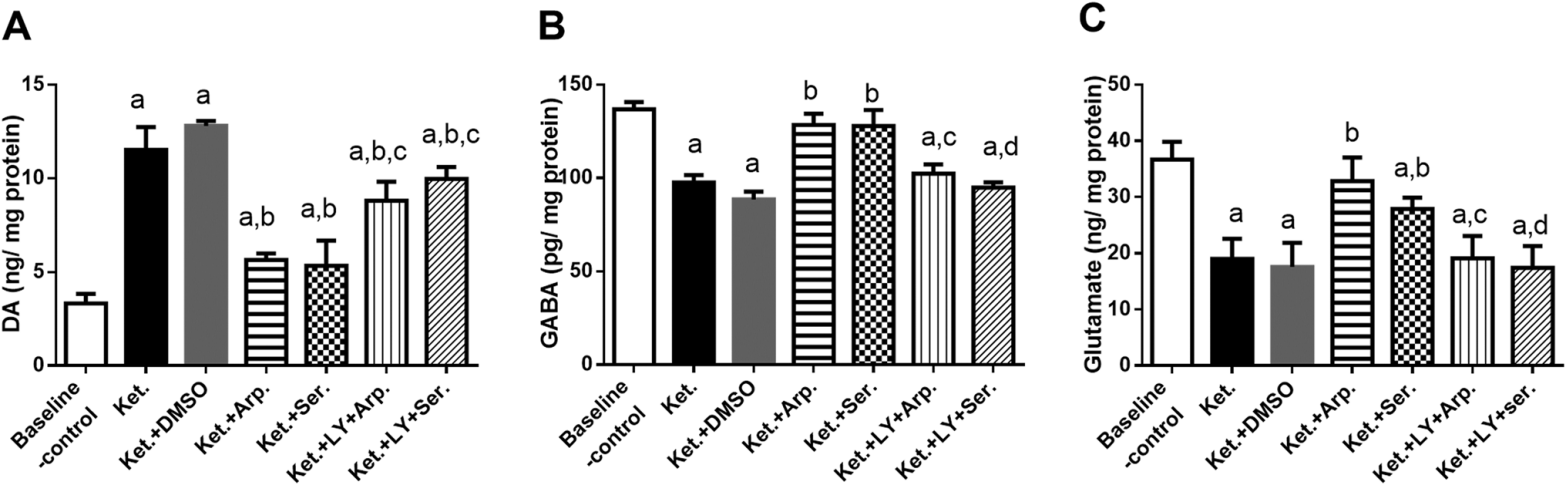
Effects of aripiprazole or sertindole treatments on DA (**A**) (F (6, 35) = 101.4, *p* < 0.0001), GABA (B) (F (6, 34) = 81.60, *p* < 0.0001), and glutamate (C) (F (6, 35) = 29.21, *p* < 0.0001) in ketamine-induced schizophrenia in rats either alone or with the PI3K inhibitor LY294002. Each bar with a vertical line represents the mean of experiments ± S.D. (*n* = 6). a vs. the baseline control group, b vs. ketamine-treated rats, c vs. the aripiprazole-treated group, and d vs. the sertindole-treated group. Statistical analyses were performed using ANOVA followed by Tukey’s post hoc test, and the criterion for statistical significance was set to *p* < 0.05

As illustrated in Fig. 10, the Ket group had a 3.5-fold higher DA level than the baseline control group. Aripiprazole and sertindole reduced the DA level by 50 and 55%, respectively, compared with the Ket group. Again, LY294002 abolished the effects of aripiprazole and sertindole on the DA level.

Next, ketamine decreased GABA and glutamate levels to 70 and 50% of the baseline control values, respectively. Aripiprazole and sertindole increased glutamate levels by 1.7- and 1.4-fold, respectively, and GABA levels by 1.3-fold compared with the Ket group. Finally, LY294002 abrogated the effects of the antipsychotics on glutamate and GABA levels (Fig. 10).

### Effects of aripiprazole and sertindole on ketamine-induced changes in GAD67 and PV expression

**Fig. 11:**
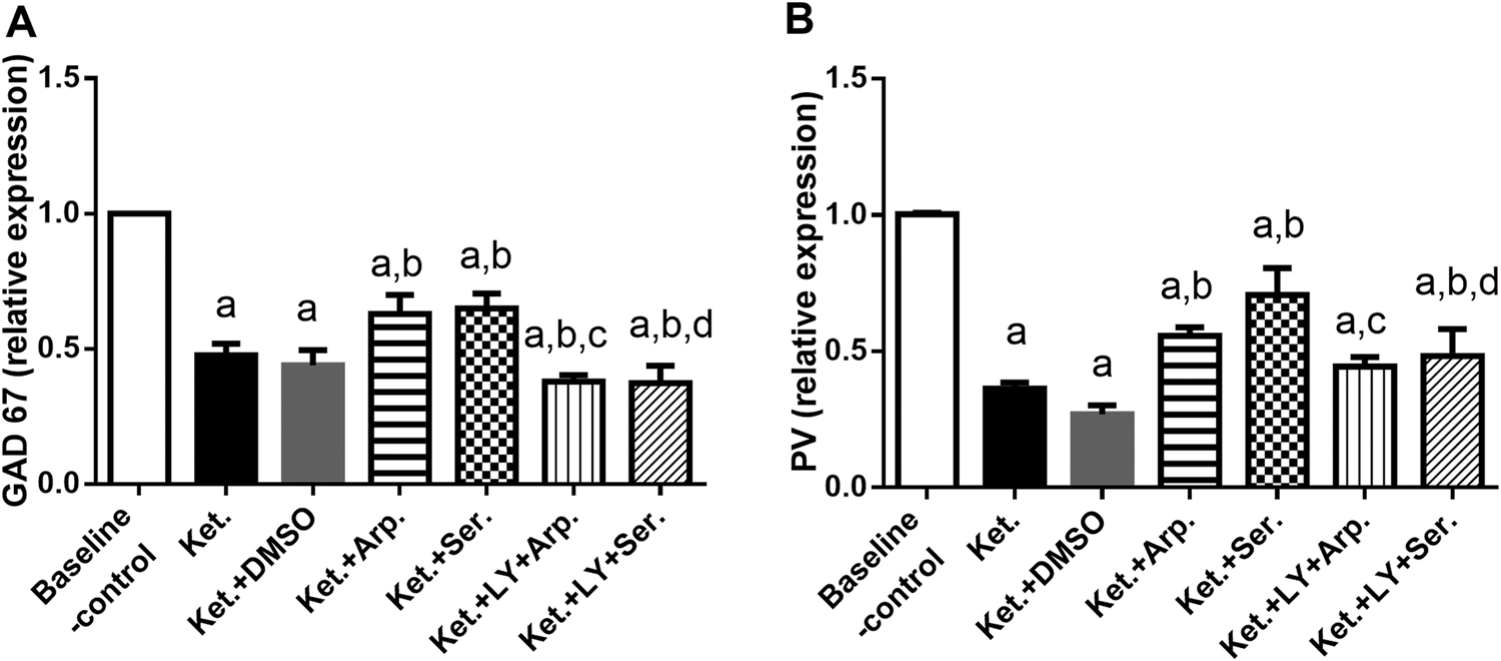
Effects of.aripiprazole or sertindole treatments on GAD 67 (**A**) (F (6, 35) = 118.1, *p* < 0.0001), and PV (**B**) (F (6, 34) = 105.6, *p* < 0.0001) in ketamine-induced schizophrenia in rats either alone or with the PI3K inhibitor LY294002. Each bar with a vertical line represents the mean of experiments ± S.D. (*n* = 6). a vs. the baseline control group, b vs. ketamine-treated rats, c vs. the aripiprazole-treated group, and d vs. the sertindole-treated group. Statistical analyses were performed using ANOVA followed by Tukey’s post hoc test, and the criterion for statistical significance was set to *p* < 0.05

Another important aspect of ketamine-induced schizophrenia was the reduction in GAD67 and PV expression by almost 56 and 63%, respectively. Aripiprazole and sertindole, respectively, increased GAD67 expression by 1.3- and 1.4-fold and PV expression by 1.7- and twofold compared with the Ket group. LY294002 inhibited the effects of the antipsychotics (Fig. 11).

### Effects of aripiprazole and sertindole on ketamine-induced changes in the NRG1/ERbB4 and PI3K signaling pathways

**Fig. 12:**
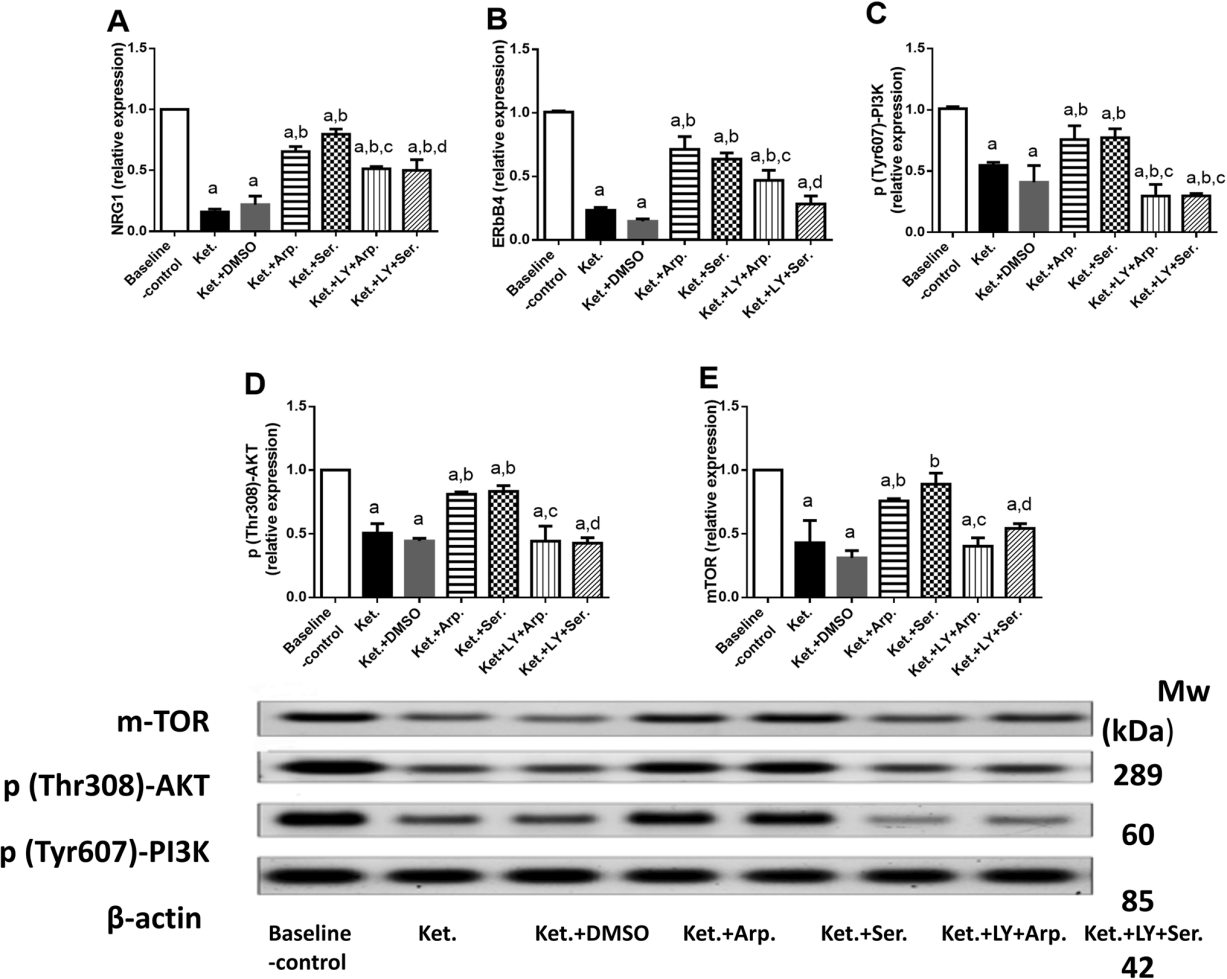
Effects of the aripiprazole and sertindole treatments on NRG1 (**A**) (F (6, 33) = 216.6, *p* < 0.0001), ErbB4 (**B**) (F (6, 35) = 167.5, *p* < 0.0001), PI3K (**C**) (F (6, 35) = 66.41, *p* < 0.0001), AKT (**D**) (F (6, 35) = 96.76, *p* < 0.0001), and mTOR (**E**) (F (6, 35) = 61.81, *p* < 0.0001) in ketamine-induced schizophrenia in rats either alone or with the PI3K inhibitor LY294002. Each bar with a vertical line represents the mean of experiments ± S.D. (*n* = 6). a vs. the baseline control group, b vs. ketamine-treated rats, c vs. the aripiprazole-treated group, and d vs. the sertindole-treated group. Statistical analyses were performed using ANOVA followed by Tukey’s post hoc test, and the criterion for statistical significance was set to *p* < 0.05

Ketamine injection downregulated NRG1 and ErbB4 mRNA expression, and P-PI3K, P-AKT, and mTOR protein expression by 85, 77, 46, 50, and 57%, respectively, as compared with the baseline control. Aripiprazole upregulated NRG1 and ErbB4 mRNA expression, and P-PI3K, P-AKT, and mTOR protein expression to 6-, 3-, 1.4-, 1.6-, and 1.7-fold, respectively, whereas sertindole increased these parameters to 5-, 2.7-, 1.4-, 1.6-, and twofold, respectively, compared with Ket group. As expected, LY294002 abolished those increases (Fig. 12).

## Discussion

This study highlights the role of the NRG1/ERbB4 and PI3K/AKT/mTOR signaling pathways in the neuroprotective effects of aripiprazole and sertindole on ketamine-induced schizophrenia in rats. The administration of subanesthetic doses of ketamine (30 mg/kg/day) for 5 consecutive days to rats is a widely used, suitable schizophrenia animal model that mimics psychotic behavior in rats and enables the study of positive, negative, and cognitive impairments (Neill et al. 2010; Chatterjee et al. 2011).

The results of our behavioral experiments are consistent with those of previous studies (Boultadakis and Pitsikas 2010; Neill et al. 2010; Bolton et al. 2012; Chindo et al. 2012; Gama et al. 2012; Ram et al. 2013; Georgiadou et al. 2014; Kandratavicius et al. 2015; Janardhanan et al. 2017; Ibrahim et al. 2022), showing that ketamine administration to rats produced schizophrenia-like behavioral changes. Herein, the marked rise in ambulation and rearing frequencies in the open field test revealed ketamine-induced hyperlocomotion. Ketamine also produced severe social deficits, as evidenced by the social interaction test, where ketamine-treated rats had significantly higher onset latency, fewer contacts with each other, and shorter interaction duration. Moreover, ketamine disrupted the rats’ recognition memory capacities assessed in the novel object recognition task. Besides, we confirmed that any differences in learning and memory were not the result of a reduced nociceptive response, as the tail flick data clearly verified that the ketamine dose was subanesthetic. We also evaluated the negative schizophrenia symptoms using the sucrose preference test. Ketamine-treated rats were anhedonic, as the significant reduction in sucrose intake showed. Treatment with either aripiprazole or sertindole ameliorated these ketamine-induced schizophrenia-like symptoms, as expected, because of their antipsychotic activity. Interestingly, LY294002, a selective PI3K inhibitor, abolished this amelioration, suggesting that PI3K signaling pathway activation is an indispensable mechanism of action of these antipsychotics.

These results were coherent with the histopathological experiments, which demonstrated that both aripiprazole and sertindole effectively preserved the cerebral cortex and hippocampus tissues and that LY294002 reduced this effect.

Many clinical and preclinical studies revealed that the brain defense systems produce reactive oxygen species (ROS) in psychiatric diseases (Che et al. 2010; Michel et al. 2011; Rao et al. 2012). Thus, oxidative damage plays a critical role in the pathophysiology of different neuropsychiatric diseases, including depression, anxiety, schizophrenia, and autism (Valko et al. 2007; Ng et al. 2008; Bouayed et al. 2009). This work showed that ketamine administration resulted in oxidative imbalance, as manifested by the reduced hippocampal GSH level and increased lipid peroxidation. These findings agree with those of previous studies that demonstrated a link between ketamine administration and oxidative stress (de Oliveira et al. 2009; Radonjić et al. 2010; Rao et al. 2012). Moreover, both aripiprazole and sertindole alleviated ketamine-induced oxidative stress in the hippocampus of rats, and the PI3K inhibitor LY294002 reduced this effect in both cases. These results indicate that aripiprazole and sertindole may exert their antipsychotic activity by eliminating ROS.

The cognitive impairments of schizophrenia are associated with GABAergic system dysfunction (Hoftman et al. 2015). Autopsy studies have shown that patients with schizophrenia had low mRNA levels of the GABAergic markers GAD67 (the 67-kDa isoform of glutamic acid decarboxylase) and PV (Glausier et al. 2014). Ketamine reduces the activity of PV interneurons by blocking NMDA receptors and thereby disinhibits cortical pyramidal neurons, including neurons that disinhibit DA neuron firing (Lodge and Grace 2009). Thus, ketamine administration has been associated with a DA neuron firing increase (El Iskandrani et al. 2015) and a glutamatergic system hypofunction (Chatterjee et al. 2015; Ahmed et al. 2018). In line with this association, we observed that ketamine reduced hippocampal GABA and glutamate levels and GAD67 and PV mRNA expression and increased DA levels in rats. Furthermore, aripiprazole and sertindole significantly reversed the ketamine-induced abnormalities in hippocampal neurotransmitters, whereas LY294002 abrogated their effects.

Previous studies have pointed out the role of NRG1 and its receptor ErbB4 in the pathophysiology of schizophrenia (Stefansson et al. 2002; Rimer et al. 2005; Dejaegere et al. 2008; Savonenko et al. 2008; Law et al. 2012; Mei and Nave 2014). The NRG1/ErbB4 signaling pathway regulates the formation and maintenance of GABAergic neuronal network activity in the cortex and hippocampus (Buonanno 2010). ErbB4 mutant mice have significantly fewer GAD67-positive and PV-positive cells (Gu et al. 2005; Neddens and Buonanno 2010), indicating synaptic dysfunction.

Recent studies have emphasized that ketamine reduces NRG1 and ERbB4 expression in different brain regions (Bian et al. 2009; Xie et al. 2020a; Grieco et al. 2021). This study found that ketamine decreased NRG1 and ErbB4 mRNA expression levels, whereas both aripiprazole and sertindole reversed these effects. Our results suggest that one of the mechanisms of these antipsychotics may be NRG1/ErbB4 signaling pathway activation. In line with this hypothesis, olanzapine, risperidone, and haloperidol restored NRG1 and ErbB4 expression levels in mouse primary hippocampal neurons treated with an NMDA antagonist (Li et al. 2016). These findings were contrary to the facts that patients with schizophrenia had high hippocampal NRG1 and ErbB4 levels (Petryshen et al. 2005; Hahn et al. 2006; Law et al. 2006) and that blocking NRG1/ErbB4 signaling improves cognitive impairment in these patients (Hasan et al. 2020). The degree of alterations in risk genes, the use of different antipsychotics, and different treatment durations may explain this inconsistency.

The PI3K signaling is a key pathway controlling cell survival, proliferation, and apoptosis. Altered PI3K signaling pathway has also been associated with schizophrenia (Enriquez-Barreto and Morales 2016). NRG1 and its receptor ErbB4 regulate PI3K/Akt signaling. The activation of ErbB4 receptors by NRG1 causes the phosphorylation of PI3K and its activation (Mei and Xiong 2008). PI3K phosphorylates and activates AKT, which results in the activation of mTOR (Polak and Hall 2009). The AKT/mTOR signaling pathway regulates protein synthesis and the actin cytoskeleton, which are crucial events for long-term memory formation and neural plasticity (Fortin et al. 2012).

The present data show that ketamine reduced hippocampal PI3K, p-AKT, and mTOR expression levels in rats, which is consistent with previous studies (Shang et al. 2007; Zuo et al. 2016; Xie et al. 2020b). In parallel, study by Chadha and Meador-Woodruff (2020) suggests that the AKT-mTOR signaling pathway is downregulated in schizophrenia brain. Moreover, excessive ROS production prevents the PI3K/AKT signaling pathway activation (Zhang et al. 2012). Considering the GSH and MDA levels in this study, we speculated that ketamine administration increased ROS production, which also inhibits the PI3K/AKT pathway. Herein, both aripiprazole and sertindole prevented ketamine-induced downregulation of PI3K, p-AKT, and mTOR expression, whereas blocking the PI3K/AKT pathway with LY294002 abolished all these effects.

In conclusion, our data demonstrate that both aripiprazole and sertindole exerted an antipsychotic activity in rats with ketamine-induced schizophrenia and that LY294002, an inhibitor of PI3K, blocked this activity. This study indicates that the antipsychotic effects of aripiprazole and sertindole are partly mediated by reduced ROS production and NRG1/ErbB4 and PI3K/AKT/mTOR signaling pathways activation. The NRG1/ERbB4 and PI3K signaling pathways may offer a new therapeutic approach to developing treatment for schizophrenia in humans, which needs to be assessed in further studies.

## Data Availability

Enquiries about data availability should be directed to the authors.
